# Frequency and imaging correlates of neuropsychiatric symptoms in Progressive Supranuclear Palsy

**DOI:** 10.1007/s00702-023-02676-9

**Published:** 2023-08-03

**Authors:** Sofia Cuoco, Sara Ponticorvo, Filomena Abate, Maria Francesca Tepedino, Roberto Erro, Renzo Manara, Gianfranco Di Salle, Francesco Di Salle, Maria Teresa Pellecchia, Fabrizio Esposito, Paolo Barone, Marina Picillo

**Affiliations:** 1 Department of Medicine, Surgery and Dentistry “Scuola Medica Salernitana”, Center for Neurodegenerative Diseases (CEMAND), University of Salerno, Neuroscience Section, Via Allende, 84081 Baronissi (Salerno), Italy; 2 Center for Magnetic Resonance Research (CMRR), Department of Radiology, University of Minnesota, 2021 6th St. SE, Minneapolis, MN 55455 USA; 3 Department of Neurosciences, Neuroradiology Unit, University of Padua, 35128 Padua, Italy; 4 Scuola Superiore Di Studi Universitari E Perfezionamento Sant’Anna, Classe Di Scienze Sperimentali, Pisa, Italy; 5 Department of Advanced Medical and Surgical Sciences, University of Campania “Luigi Vanvitelli”, 80138 Naples, Italy

**Keywords:** MRI, Neuropsychiatric symptoms, NPI, Progressive Supranuclear Palsy

## Abstract

Neuropsychiatric symptoms are intrinsic to Progressive Supranuclear Palsy (PSP) and a spoonful of studies investigated their imaging correlates. Describe (I) the frequency and severity of neuropsychiatric symptoms in PSP and (II) their structural imaging correlates. Twenty-six PSP patients underwent Neuropsychiatric Inventory (NPI) and brain 3D T1-weighted MRI. Spearman’s rho with Bonferroni correction was used to investigate correlations between NPI scores and volumes of gray matter regions. More than 80% of patients presented at least one behavioral symptom of any severity. The most frequent and severe were depression/dysphoria, apathy, and irritability/lability. Significant relationships were found between the severity of irritability and right pars opercularis volume (p < 0.001) as well as between the frequency of agitation/aggression and left lateral occipital volume (p < 0.001). Depression, apathy, and irritability are the most common neuropsychiatric symptoms in PSP. Moreover, we found a relationship between specific positive symptoms as irritability and agitation/aggression and greater volume of the right pars opercularis cortex and lower volume of the left occipital cortex, respectively, which deserve further investigations.

## Introduction

Progressive Supranuclear Palsy (PSP) is a rare neurodegenerative disease characterized by postural instability, ocular motor dysfunction, akinesia, and cognitive as well as neuropsychiatric disturbances (Höglinger et al. 2017; Picillo et al. 2018). The diverse combination of core clinical features is determinant for the attribution of the clinical phenotype of the disease. While PSP Richardson’s syndrome (PSP-RS) is the most common clinical phenotype, other distinct variants (vPSP) have been described, each featuring a specific predominant symptom (Höglinger et al. 2017; Grimm et al. 2019).

Irrespective of the phenotype, neuropsychiatric symptoms are intrinsic to PSP and strongly correlate with cognitive deterioration determining a dramatic impact on quality of life and daily functioning (Litvan et al. 1996; Gerstenecker et al. 2013). The distribution of neuropsychiatric symptoms in PSP is heterogeneous (Litvan et al. 1996; Gerstenecker et al. 2013; Yatabe et al. 2011; Bower et al. 2021; Ječmenica-Lukić et al. 2018; Flavell and Nestor 2022). Previous studies showed that apathy, aberrant motor behavior, and disinhibition are the most common neuropsychiatric disturbances followed by psychosis and depression (Litvan et al. 1996; Yatabe et al. 2011). More recent evidence would suggest the most important determinants of increased Neuropsychiatric Inventory (NPI) total score in PSP are apathy and depression (Ječmenica-Lukić et al. 2018).

A few studies investigated imaging correlates of neuropsychiatric symptoms in PSP patients (Josephs et al. 2011; Lansdall et al. 2017, 2018; Urso et al. 2022). An association between apathy and basal ganglia atrophy, including caudate, putamen, and thalamus, has been reported by different studies (Josephs et al. 2011; Lansdall et al. 2017).

Herein, we sought to describe: (I) the frequency and severity of neuropsychiatric symptoms screened with the NPI in non-demented PSP patients and (II) their structural imaging correlates.

## Methods

Consecutive patients with a diagnosis of PSP according to the Movement Disorders Society clinical criteria were enrolled at the Center for Neurodegenerative Diseases of the University of Salerno, Italy, at the movement disorder section, between November 2015 and April 2019 [1, 3]. Only patients qualifying for a degree of diagnostic certainty of probability were included in the present study. Caregivers completed the NPI with reference to the patients, assessing frequency and severity of 12 behavioral areas, including delusions, hallucinations, agitation/aggression, depression/dysphoria, anxiety, euphoria, apathy, disinhibition, irritability/lability, aberrant motor behavior, and sleep and eating disorders. Specifically, frequency was measured with a 5-point Likert scale (0 = never, 1 = rarely, 2 = sometimes, 3 = frequently, and 4 = almost constantly) and severity was measured with a 3-point Likert scale [1 = mild (do not disturb the patient); 2 = moderate (disturbing the patient); 3 = severe (require the administration of drugs; they are very disturbing for the patient)]. The emotional or psychological stress of the caregiver section was measured with a 6-point Likert scale (0 = nobody, 1 = minimum, 2 = mild, 3 = moderate, 4 = severe, and 5 = serious). The NPI section regarding caregiver’s stress was filled in but was not used in the correlation analysis with imaging data as it primarily concerns the caregiver and not the patient. According to the NPI instructions, caregivers were asked to refer to situation of the patient in the 6 weeks preceding the interview (the last month and a half). We chose the NPI as it is a solid instrument to evaluate behavioral disturbances in a wide range of neurodegenerative diseases and has been administered to PSP patients in several previous studies (Cummings et al. 1994*;* Litvan et al. 1996, 1998; Aarsland et al. 2001). Severity of disease was evaluated with the Progressive Supranuclear Palsy rating scale (PSP-rs) (Golbe and Ohman-Strickland 2007) and cognitive dysfunction with the MOntreal Cognitive Assessment (MOCA) (Santangelo et al. 2014). Patients scoring less than 13 with the MOCA were excluded from the present study as we intended to focus the present study on the neuropsychiatric characterization of non-demented PSP.

Magnetic Resonance Imaging (MRI) data were acquired on a 3 T MRI scanner (MAGNETOM Skyra, Siemens, Erlangen, Germany) operated with a 20-channel head and neck coil. A 3D anatomical T1-weighted (T1w) Magnetization Prepared RApid Gradient Echo (MPRAGE) sequence was acquired with repetition time (TR) = 2400 ms and echo time (TE) = 2.25 ms, spatial resolution = 1 × 1 × 1 mm3, matrix size = 256 × 256, anterior–posterior phase encoding direction, and generalized autocalibrating partially parallel acquisitions (GRAPPA) factor of 2 in phase encoding. MPRAGE images were processed using FreeSurfer version 6.0 (https://surfer.nmr.mgh.harvard.edu/). Raw T1w images of all subjects were imported in FreeSurfer and submitted to the standard structural image preprocessing and reconstruction pipeline via the “recon-all" command (for a detailed description of this procedure, please see https://surfer.nmr.mgh.harvard.edu/fswiki/recon-all) (Dale and Sereno 1993; Dale et al. 1999; Fischl et al. 1999; Fischl and Dale 2000; Fischl et al. 2001, 2002). For each subject, the volumes of 68 cortical regions, according to the Desikan–Killiany atlas (Desikan et al. 2006) and 16 subcortical regions were calculated.

The study was approved by the local Ethics Committee and all patients signed informed consent.

Comparison of presence of behavioral symptoms between groups was assessed using the Chi-squared test. Spearman’s correlation with Bonferroni correction was used to explore correlations among neuropsychiatric symptoms frequency. The relationship between MRI cortical and subcortical volumes and neuropsychiatric symptoms was evaluated by Spearman’s correlations with Bonferroni correction for multiple comparisons on the number of regions (*12 neuropsychological symptoms related to* 68 and 16 regions for cortex and subcortical areas, respectively). Particularly, correlation was calculated between frequency of symptoms and regional volumes in all patients and between severity of symptoms and regionals volumes only in the subgroup of patients presenting symptoms (frequency > 0). Correlations were considered strong with coefficients > 0.70 and moderate with coefficients between 0.30 and 0.70.

We divided the sample in patients with disease duration ≤ 2 years, patients with disease duration > 2 years but ≤ 4 years, and patients with disease duration ≥ 5 years. We compared the presence/absence of symptoms between the three subgroups with MANOVA with Bootstrap method using clinical variables that differ significantly between subgroups as covariates.

All analyses were performed in SPSS for Windows, version 23.0 and MATLAB 2019a.

## Results

Twenty-six PSP patients [13 men/13 women; 14 PSP-RS/12 vPSP (2 PSP with predominant frontal presentation, 2 PSP with predominant parkinsonism, 5 PSP with predominant progressive gait freezing, 3 PSP-corticobasal syndrome)] with a mean (± standard deviation) age equal to 68.92 ± 5.93, a mean education equal to 9.12 ± 5.86, and a mean disease duration equal to 3.15 ± 1.74 were included The mean MOCA and PSP-rs scores were 17.54 ± 4.07 and 37 ± 14.98, respectively.

In the whole sample, 80.8% of PSP patients presented at least one behavioral symptom of any severity. No differences were detected in the distribution of behavioral symptoms between sexes (p = 0.619) or PSP phenotypes (p = 0.192).

The frequency and severity of neuropsychiatric symptoms as reported by the NPI are shown in Table 1. The most frequent symptoms determining the greatest caregiver’s stress were depression/dysphoria (frequency: 76.9%; severity: 1.23 ± 0.81; caregiver’s stress: 1.08 ± 1.16), apathy (frequency: 65.3%; severity: 1.15 ± 1.00; caregiver’s stress: 0.92 ± 1.19), and irritability/lability (frequency: 46.1%; severity: 0.65 ± 0.79; caregiver’s stress: 0.85 ± 1.15). Delusions and hallucinations were reported by a minority of patients and determined negligible or no caregiver’s stress (frequency: 7.7%, severity: 0.08 ± 0.27 for both, caregiver’s stress: 0.08 ± 0.39 for delusions and 0.0 ± 0.0 for hallucinations).

**Table 1:** The frequency and severity of neuropsychiatric symptoms as reported by the Neuropsychiatric Inventory

	Frequency		Severity		Caregiver’s stress	
	%	Mean ± SD	%	Mean ± SD	%	Mean ± SD
**Delusions**	*Never* = *92.3%* *Rarely* = 7.7% *Sometimes* = 0% *Frequently* = 0% *Constantly* = 0% **Total = 7.7%**	0.08 ± 0.27	Absent = 92.3% Mild = 7.7% Moderate = 0% Severe = 0%	0.08 ± 0.27	Nobody = 96.2% Minimum = 0% Mild = 3.8% Moderate = 0% Severe = 0% Serious = 0%	0.08 ± 0.39
**Hallucinations**	*Never* = *92.3%* *Rarely* = 7.7% *Sometimes* = 0% *Frequently* = 0% *Constantly* = 0% **Total = 7.7%**	0.08 ± 0.27	Absent = 92.3% Mild = 7.7% Moderate = 0% Severe = 0%	0.08 ± 0.27	Nobody = 100% Minimum = 0% Mild = 0% Moderate = 0% Severe = 0% Serious = 0%	0.00 ± 0.00
**Agitation/aggression**	*Never* = *77%* *Rarely* = 3.8% *Sometimes* = 11.5% *Frequently* = *7.7%* *Constantly* = *0%* **Total = 23%**	0.50 ± 0.99	Absent = 76.9% Mild = 11.5% Moderate = 11.5% Severe = 0%	0.35 ± 0.68	Nobody = 80.8% Minimum = 7.7% Mild = 0% Moderate = 11.5% Severe = 0% Serious = 0%	0.42 ± 0.98
**Depression/dysphoria**	*Never* = *23.1%* *Rarely* = *11.5%* *Sometimes* = *38.5%* *Frequently* = *23.1%* *Constantly* = *3.8%* **Total = 76.9%**	1.79 ± 1.18	Absent = 19.2% Mild = 42.3% Moderate = 34.6% Severe = 3.8%	1.23 ± 0.81	Nobody = 38.5% Minimum = 34.6% Mild = 11.5% Moderate = 11.5% Severe = 3.8% Serious = 0%	1.08 ± 1.16
**Anxiety**	*Never* = *69.3%* *Rarely* = *3.8%* *Sometimes* = *19.2%* *Frequently* = *7.7%* *Constantly* = *0%* **Total = 30.7%**	0.65 ± 1.05	Absent = 69.2% Mild = 7.7% Moderate = 23.1% Severe = 0%	0.54 ± 0.85	Nobody = 69.2% Minimum = 11.5% Mild = 15.4% Moderate = 3.8% Severe = 0% Serious = 0%	0.54 ± 0.90
**Euphoria**	*Never* = *84.7%* *Rarely* = *3.8%* *Sometimes* = *11.5%* *Frequently* = *0%* *Constantly* = *0%* **Total = 15.3%**	0.27 ± 0.66	Absent = 84.6% Mild = 11.5% Moderate = 3.8% Severe = 0%	0.19 ± 0.49	Nobody = 88.5% Minimum = 3.8% Mild = 3.8% Moderate = 3.8% Severe = 0% Serious = 0%	0.23 ± 0.71
**Apathy**	*Never* = *34.7%* *Rarely* = *11.5%* *Sometimes* = *19.2%* *Frequently* = *11.5%* *Constantly* = *23.1%* **Total = 65.3%**	1.77 ± 1.60	Absent = 34.6% Mild = 23.1% Moderate = 34.6% Severe = 7.7%	1.15 ± 1.00	Nobody = 53.8% Minimum = 15.4% Mild = 19.2% Moderate = 7.7% Severe = 3.8% Serious = 0%	0.92 ± 1.19
**Disinhibition**	*Never* = *88.5%* *Rarely* = *0%* *Sometimes* = *11.5%* *Frequently* = *0%* *Constantly* = *0%* **Total = 11.5%**	0.23 ± 0.65	Absent = 88.5% Mild = 3.8% Moderate = 7.7% Severe = 0%	0.19 ± 0.56	Nobody = 88.5% Minimum = 3.8% Mild = 0% Moderate = 7.7% Severe = 0% Serious = 0%	0.27 ± 0.82
**Irritability/lability**	*Never* = *53.9%* *Rarely* = *11.5%* *Sometimes* = *15.4%* *Frequently* = *19.2%* *Constantly* = *0%* **Total = 46.1%**	1.00 ± 1.23	Absent = 53.8% Mild = 26.9% Moderate = 19.2% Severe = 0%	0.65 ± 0.79	Nobody = 57.7% Minimum = 15.4% Mild = 11.5% Moderate = 15.4% Severe = 0% Serious = 0%	0.85 ± 1.15
**Aberrant motor behavior**	*Never* = *80.8%* *Rarely* = *7.7%* *Sometimes* = *3.8%* *Frequently* = *7.7%* *Constantly* = *0%* **Total = 19.2%**	0.38 ± 0.89	Absent = 80.8% Mild = 11.5% Moderate = 7.7% Severe = 0%	0.27 ± 0.60	Nobody = 80.8% Minimum = 7.7% Mild = 3.8% Moderate = 3.8% Severe = 3.8% Serious = 0%	0.42 ± 1.02
**Sleep disorders**	*Never* = *65.4%* *Rarely* = *7.7%* *Sometimes* = *15.4%* *Frequently* = *7.7%* *Constantly* = *3.8%* **Total = 34.6%**	0.77 ± 1.21	Absent = 65.4% Mild = 15.4% Moderate = 7.7% Severe = 11.5%	0.69 ± 1.15	Nobody = 76.9% Minimum = 15.4% Mild = 0% Moderate = 3.8% Severe = 3.8% Serious = 0%	0.42 ± 0.98
**Eating disorders**	*Never* = *80.9%* *Rarely* = *7.7%* *Sometimes* = *3.8%* *Frequently* = *3.8%* *Constantly* = *3.8%* **Total = 19.1%**	0.42 ± 1.02	Absent = 80.8% Mild = 11.5% Moderate = 7.7% Severe = 0%	0.29 ± 0.60	Nobody = 88.5% Minimum = 0% Mild = 3.8% Moderate = 7.7% Severe = 0% Serious = 0%	0.31 ± 0.88

Bold indicate the percentage of total frequency of symptoms

SD, standard deviation

As for the correlation of frequency of neuropsychiatric symptoms between each other, depression was significantly related to apathy (rho = 0.67, p < 0.001), disinhibition was significantly related to euphoria (rho = 0.83, p < 0.001) and apathy was significantly related to sleep disorders (rho = 0.59, p = 0.002). No other associations between neuropsychiatric symptoms were detected.

As for the correlation between neuropsychiatric symptoms and imaging data, Spearman’s correlation showed a significant relationship between the severity of irritability and right pars opercularis volume (rho = 0.856, p < 0.001) as well as between the frequency of agitation/aggression and left lateral occipital volume (rho =  − 0.622, p < 0.001). No other significant correlations were found.

**Fig. 1:**
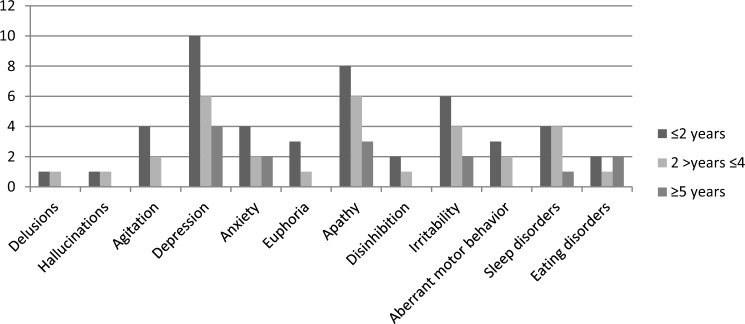
The distribution of symptoms according to disease duration

Figure 1 reports the distribution of symptoms according to disease duration. We found no significant differences between subgroups in terms of frequency of neuropsychiatric symptoms, using PSP rating scale as covariate.

## Discussion

Herein, we confirmed that the majority of PSP patients report neuropsychiatric disturbances (Gerstenecker et al. 2013; Santangelo et al. 2018). Consistent with available evidence, we found that depression/dysphoria, apathy, and irritability/lability were the most common and severe, while delusions and hallucinations were seldom reported and had a negligible impact on caregiver’s stress (Gerstenecker et al. 2013). Previous studies showed that in PSP, the neuropsychiatric profile is dominated by negative symptoms and that apathy is the most common (Litvan et al. 1996; Aarsland et al. 2001; Cordato et al. 2006). However, in our cohort, depression/dysphoria was more frequent than apathy (76.9% and 65.3%, respectively). The different instruments used and the greater confidence of the caregiver with the concept of depression may in part account for such subtle discrepancy with previous evidence (Menza et al. 1995; Millar et al. 2006; Schrag et al. 2010; Cuoco et al. 2021).

Differently from Gerstenecker et al., positive symptoms as agitation, disinhibition, and eating disorders were reported by less than a third of our cohort and received lower scores in terms of severity and caregiver’s stress (Gerstenecker et al. 2013). As half of our cohort was represented by vPSP, we could not identify different behavioral profiles in relation to PSP phenotype. Considering that previous studies were totally focused on PSP applying the previous set of criteria focused on Richardson’s syndrome, the diverse composition of the study cohort may thus explain differences with the previous data (Gerstenecker et al. 2013; Aarsland et al. 2001). Of note, a recent study by Bower et al. reported that PSP-Speech/Language (PSP-SL) variant presents lower depression frequency and scores compared to the other variants (Bower et al. 2021).

In line with previous evidence, we confirm a significant relationship between apathy and depression, two negative symptoms that can co-occur in a variety of neurodegenerative diseases (Baba 2018). Similarly, the correlation between disinhibition and euphoria was not surprising. In fact, both such symptoms are part of the hyperactivity syndrome related to prefrontal cortex involvement (Tascone and Bottino 2013; Nagata et al. 2016). On the other hand, ours is the first study reporting a link between apathy and reported sleep disorders in PSP, although such association was already reported in studies on patients with Parkinson’s disease where apathy was related to longer rapid eye movement (REM) sleep latency and more frequent periodic limb movements in the REM phase as well as with the REM sleep ratio, the apnea–hypopnea index and the oxygen deficit index (Barber et al. 2018; Chao et al. 2021). Of note, 86% of our patients reporting sleep disorders complained about sleep agitation. However, we failed to administer specific sleep questionnaires or assessments, and thus, our data are to be considered preliminary and need further investigations with regard to this aspect.

When analyzing the correlation between behavioral symptoms and the volume of the subcortical and cortical structures, we found that the severity of irritability was positively related to the right pars opercularis volume. Such surprising association may be explained considering available evidence from imaging studies in psychiatric diseases (Yamasaki et al. 2010; Hajek et al. 2013a, b; Roberts et al. 2017; Inhóf et al. 2019). In line with previous data (Sellami et al. 2018), our study supports that clinical overlap between PSP and primary psychiatric conditions may be mediated by the alteration of common structures involved in large-scale networks mainly at a cortical level (Rogozinski et al. 2022). Indeed, the role of the pars opercularis in the inferior frontal gyrus within both social and cognitive networks has been consistently reported in several studies analyzing observation of facial expressions, action observation, facial imitation, and intention understanding (Iacoboni et al. 1999; Johnson-Frey et al. 2003; Dapretto et al. 2006). As such, the right pars opercularis cortex is a hub within the circuits regulating inhibitory functioning, thus the ability to inhibit a preplanned motor response, along with other frontal and striatal regions (Chambers et al. 2009; Aron et al. 2014). Volume reduction of the right pars opercularis has been linked with increased severity of social communication problems within the Autism Spectrum Disorders (Yamasaki et al. 2010). Complementary, volume increase in the inferior frontal gyrus was shown to be a marker of susceptibility for development of bipolar disorder (Matsuo et al. 2012; Hajek et al. 2013a, b; Hajek et al. 2015; Sarıçiçek et al. 2015; Roberts et al. 2017). Overall alterations in the activation of the inferior frontal gyrus with a subsequent hypersensitivity to stimuli has been described in bipolar disorder in relation to the maniacal or euthymic phase (Selvaraj et al. 2012; Hajek et al. 2013a, b). Finally, a study analyzing imaging correlates of behavioral addictions reported a relationship between loss of control and irritability and greater right pars opercularis volume. Specifically, the authors discussed that the increased gray matter measures of this structure might be explained with the extended effort to control for the impulsive behavior in addiction (Inhóf et al. 2019). Such body of evidence from psychiatric conditions would suggest a role for the right pars opercularis in the modulation of inhibitory functioning within both emotional and motor circuits.

We also found a significant negative correlation between the frequency of agitation/aggression and the left lateral occipital volume. Although aggressive behaviors have been linked to frontal cortex in subjects with overt behavioral diseases (Blair and Lee 2013), other studies on young drinkers have linked reduced gray matter in the occipital cortex to the generation of violent and antisocial behavior (Bertsch et al. 2013; Charpentier et al. 2016). Furthermore, reduced cortical thickness in the right pericalcarine cortex has been linked to the search for strong sensations and risky behaviors in healthy young adults (Holmes et al. 2016; Miglin et al. 2019). Indeed, visual perception is crucial for the processing of facial expression, which serves to develop empathy. In a previous study, we found a significant correlation between visuo-spatial abilities and retinal layers in PSP suggesting the existence of a mutual relationship between posterior cognitive function and retinal structure (Picillo et al. 2022). Our hypothesis is that patients with agitation/aggression may have altered visual perception associated with lower occipital volumes.

Expanding our knowledge on behavioral disorders in PSP is crucial for both research and clinical purposes. Understanding the pathogenesis of neuropsychiatric disturbances in terms of involved brain areas and circuits may open new venues in the pharmacological and non-pharmacological treatment of neuropsychiatric symptoms (Vanacore and Canevelli 2019). Increasing clinicians’ awareness and ability to deal with behavioral disturbances may have a potential impact on quality of life of both patients and caregivers (Kim et al. 2021).

Our study found that two positive behavioral symptoms, such as irritability and aggression, are related with cortical structures in PSP. These findings strengthen the role between behavioral disturbances and motor control and suggest a role for the social brain in the inhibitory control of both emotional and motor response. In line with previous data (Sellami et al. 2018), our study supports that clinical overlap between PSP and primary psychiatric conditions may be mediated by the alteration of common structures involved in large-scale networks.

In terms of frequency of neuropsychiatric disturbances according to different groups of disease duration, we failed to show a peculiar distribution pattern of neuropsychiatric involvement according to time since the onset of disease. However, we acknowledge the present study which was not specifically designed to investigate this aspect.

We are aware our study has limitations. First, since we intended to focus on the behavioral characterization of non-demented PSP, we only considered patients with MOCA ≥ 13. As a matter of fact, there is no published MOCA cutoff to define dementia in PSP. Published MOCA cutoff to distinguish between mild cognitive impairment (MCI) and dementia does not refer to PSP population (Ilardi et al. 2023). Thus, we believe that such cutoff cannot simply be translated to PSP. On the other hand, in our experience, an MOCA ≥ 13 can help in focusing the interview on non-demented PSP patients. Furthermore, although it can be considered an asset having 26 PSP patients with behavioral characterization and imaging data, we recognize that our data need to verify in larger samples.

In conclusion, we demonstrated that depression/dysphoria, apathy, and irritability/lability are the most common neuropsychiatric symptoms in non-demented PSP patients. Our data also suggest a relationship between specific positive symptoms as irritability and agitation/aggression and greater volume of the right pars opercularis cortex and lower volume of the left occipital cortex, respectively, which deserve further investigations.

## Data Availability

We confirm that we have read the Journal’s position on issues involved in ethical publication and affirm that this work is consistent with those guidelines. The data have not been previously presented orally or by poster at scientific meetings.
